# Academic medical centres in the Netherlands: muddling through or radical change?

**DOI:** 10.3389/fpubh.2023.1252977

**Published:** 2024-01-04

**Authors:** Ester M. M. Cardinaal, Martijn J. H. Tjan, Patrick P. T. Jeurissen, Hubert Berden

**Affiliations:** ^1^Radboud University Medical Centre, Nijmegen, Netherlands; ^2^Radboud Institute of Health Sciences (RIHS), Nijmegen, Netherlands

**Keywords:** academic medical centre, organisation, institutional complexity, radical change, governance, incrementalism, the Netherlands

## Abstract

**Introduction:**

Academic medical centres (AMCs) are designed to perform multiple tasks within a single organisation. This institutional complexity gives rise to intricate governance challenges and promotes incrementalism and muddling.

**Method:**

In this study, we hypothesised that radical change could provide a solution to the current incrementalism and we explored the conditions under which such changes could or could not be achieved.

**Results:**

We conducted unstructured interviews with various high-level stakeholders and identified issues that negatively affected the governance of Dutch AMCs, which include: 1) negative undercurrents and unspoken issues due to conflicts of interests, 2) organisational complexity due to relationships with a university and academic medical specialists, 3) lack of sufficient government direction, 4) competition between AMCs due to perverse systemic incentives, 5) different interests, focus, and organisational culture, 6) concentration of care, which does not always lead to enhanced quality and efficiency as the provision of less complex care is of utmost importance for education and research, 7) the infeasibility of public and regional functions of an AMC, 8) the inefficiency of managing three core tasks within the same organisation and, 9) healthcare market regulation.

**Discussion:**

Our hypothesis that radical change offers a solution to the current incrementalism in AMCs could not be adequately explored. Indeed, our exploration of the conditions under which radical change could potentially take place revealed that there are factors currently at play that make a substantive conversation between stakeholders about radical change difficult, if not impossible. The results also show that the government is in a position to take the lead and create conditions that foster mutual trust and common interests among AMCs, as well as between AMCs and other hospitals.

## 1 Introduction

Between 1983 and 2007, academic medical centres (AMCs) were established in the Netherlands. The distinctive feature of AMCs, which sets them apart from other Dutch hospitals in the country, lies in their integration of patient care, research, training, and education within a single organisation. The Health Insurance Act (“Zorgverzekeringswet”), which has been in force since 2006 (1) is based on the principle of market forces, which led to a discussion on how the public functions of an AMC should be managed and regulated. Because AMCs fulfil the three public functions of tertiary care, scientific research, and medical education and training, in addition to their hospital function, the health minister has emphasised that AMCs differ from top clinical and general hospitals (2) justifying the allocation of specific funding to support these public functions. In this construct, collaboration is crucial, and both AMCs and other hospitals are expected to outdo themselves and put patient interests first, with a keen focus on balancing quality, accessibility, and affordability (3). Since their establishment in the 1980s, politicians and other stakeholders have raised questions about the effectiveness of AMCs, and critics question whether their specific funding is justified compared with other hospitals. In other words, they questioned the unique position of AMCs. For more than 40 years, the unique position of AMCs has been questioned and they have been blamed for a lack of transparent accountability (4). This is compounded by the complex interrelationships between the government, insurers, AMCs, medical faculties and professional interest groups as well as the fact that no (or only minor) changes have been made for decades, as evidenced by the history of the AMCs. In our research, we characterise this as muddling through. Charles Lindblom introduced the term “muddling through,” which he later articulated as incrementalism (5, 6) referring to the decision-making process as a series of small, mostly intuitive changes. In contrast, large, carefully planned changes make incrementalism evolutionary, rather than revolutionary. Today, incrementalism still influences empirical research and theoretical debates (7). In this study, we hypothesised that radical change could provide a solution to the current incrementalism and we explored the conditions under which such changes could or could not be achieved.

### 1.1 Theoretical framework

Dutch AMCs have been the subject of public debate since their inception. This debate questions their unique position and effectiveness. In recent years, AMCs have failed to make major changes that could silence the discussions. Therefore, in our research, we contrast the current so-called incrementalism (muddling through) with radical change.

#### 1.1.1 Incrementalism

Incrementalism refers to what Charles Lindblom introduced in 1959 with the term “muddling through,” which he later articulated as incrementalism (5, 6). He referred to the decision-making process as a series of small, mostly intuitive changes. Lindblom argued that in Western democracies, public administrators and policy analysts generally limit themselves to incremental or marginal adjustments in policies. According to him, the driving motivation is not to simplify challenges but they aspire to contribute something meaningful during their time in office. Lindblom concludes that the policies of public organisations are almost entirely incremental; changes in policy almost never involve radical change. In 2011, Rothmayer-Allison et al. conducted a comprehensive study on the current relevance of incrementalism in public policy and public administration. Their research shows that Lindblom’s incrementalism is still relevant today (7).

#### 1.1.2 Radical change

Radical organisational change involves letting go of existing organisational structures and transformation to other structures (8). Unlike convergent change, which involves minor adjustments, radical change requires letting go of an existing situation and creating a new one better suited to current challenges (9). In their article, Chreim et al. conclude that it is difficult to make radical changes in organisations and systems in general and in those of healthcare systems in particular (10). Healthcare systems are characterised by the presence of diverse goals and multiple stakeholders with different interests. Radical change consists of changes in values, structures and practices and in order for multiple stakeholders from a healthcare system to agree on the form and content of radical change, several facilitating factors must be present (11, 12). According to Greenwood and Hinings (13) there is an increasing need for organisational change and a growing focus on radical change.

## 2 Methods

We began with an overview of the historical, political and legal landscapes in which Dutch AMCs operate. Following this, we conducted unstructured interviews with expert stakeholders who possess comprehensive knowledge regarding healthcare management and polices in the Netherlands with a specific focus on AMC’s. To the best of researchers’ knowledge, no prior research has been conducted on this topic. This study is an initial exploration of themes around the topic. Therefore, a small research population was chosen, meaning that not all perspectives (stakeholders) around this topic were included in the study. The purpose of an unstructured interview was to have a free conversation with the respondents. Since there is little scientific data available on this topic, the intention was to determine the interview questions during the course of the interview. Furthermore the unstructured setting was intended to create an environment in which respondents felt that they could control the interaction, which might make them more open to giving in-depth answers (14, 15). Participants were invited to participate in the interview by initiating the discussion with a probing opening question, inquiring whether the complexity of AMC governance would decrease in the scenario of one AMC, opposed to the current seven. The interviewers monitored the boundaries of the interview topic with questions as mentioned in Supplementary File 3. This approach was aimed at gaining insider perspectives and expanding the limited current understanding of the topic. To ensure methodological rigour, we adhered to the COREQ (Consolidated criteria for reporting qualitative research) checklist (Supplementary File 1) (16). Two authors (EC and MT) conducted unstructured interviews. Participants were selected based on purposive sampling (17). The selection process aimed to ensure that the respondents were representative of the key strategic issues pertaining to AMCs. Respondents (*n* = 7) from different organisations with different functions and perspectives on the healthcare landscape were asked to broaden the scope of this study (Supplementary File 2). Interviews were conducted between October 2020 and December 2020. Interviews were preferably conducted in-person (*n* = 4). However, some interviews were performed using video conferencing due to travel distance, and personal preferences arising from the COVID-19 pandemic (n = 2). One participant was interviewed via telephone because video conferencing was not possible. Prior to starting the interviews, written informed consent was obtained and participants were given a brief overview of the study (see Supplementary File 3). Subsequently, the audio recording was initiated. To reduce the risk of technical failure, two audio recorders were used for each interview. The duration of the interviews varied between 45 and 60 min. All the interviewees were offered copies of their transcripts.

As we were looking for the opinions and experiences of high-level stakeholders regarding AMCs’ governance, we used an inductive approach by applying thematic analysis, in the data analysis (18). The themes were determined by the data we obtained from the interviewees. The interviews were transcribed using ATLAS t.i. 8.4.20 (19). The transcripts were analysed in which data collection and analysis took place simultaneously to facilitate the refinement of subsequent interviews (e.g., explore areas not previously covered). Codes were created using an inductive coding strategy. Codes were thematically analysed, wherein loose codes were grouped into subthemes and overarching themes (20). This helped make connections despite the large amount of raw data. For each subtheme and overarching theme, quotations were marked in the transcripts to elaborate on the context or meaning. A total of 97 codes were derived from seven transcripts. These codes were grouped into 14 sub-themes and into nine overarching themes to establish connections between the different codes (Supplementary File 4). Coding was prepared by one author (MT) and feedback was provided by a second author (EC). The same method was applied to create subthemes as overarching themes, thereby minimising the risk of bias among the coders (21). We had the themes determined by the data we got from the interviews. Inherent in the systematics of thematic analysis is the possibility that not all themes were covered. Nevertheless, the authors agree that key themes have emerged in this study. Participants were invited to provide feedback on the transcription. None of the interviewees made any changes.

## 3 Results

We hypothesised that radical change might offer a solution to the current incrementalism and explored the conditions under which such changes could or could not be achieved. Our hypothesis that radical change offers a solution to the current incrementalism in AMCs could not be adequately explored. Our exploration of the conditions under which radical change could take place revealed that there are currently factors at play that make implementing radical reforms in healthcare difficult, if not impossible.

### 3.1 Historical context

Until the end of the 19th century, most patient care in The Netherlands took place at home, at least for those who could afford it. Over time, medical care outside the home became increasingly accepted. This development culminated in the establishment of the first private hospitals. In the late 19th century, some private hospitals expanded patient care to include teaching and research. This created the forerunners of AMCs. In the 20th century, demand, supply and costs in Dutch healthcare increased enormously, leading to the beginning of the reorganisation of Dutch hospital care in the 1970s. For Dutch AMCs, this meant giving them by law the status of independent legal entities (22). With this, the law linked AMCs to medical faculties for education and research, giving AMCs a special position in the Dutch hospital landscape (23). Nowadays The Netherlands can be defined a decentralised unitary state with approximately 17,5 million inhabitants, in which health policy is decided at the national level with some delegation of health system management to local government (provinces and municipalities). The health system is characterised by a mix of regulated competition and market-oriented, incentive-based health care (24). Dutch hospital care is divided between academic medical centres, top clinical hospitals and general hospitals (Table 1). AMCs are large hospitals with a leading position in highly complex patient care, scientific research, training and education. Top clinical hospitals provide basic care as well as care requiring specific specialised facilities, they offer training places to medical specialists and often participate in scientific research. General hospitals are regional hospitals that provide mainly basic care and are relatively small and therefore usually do not have specialised teams for many types of diseases. Around these hospital groups, the landscape also includes outpatient clinics, specialised hospitals and independent treatment centres.

**Table 1:** Key figures AMCs, top clinical hospitals, general hospitals (figures rounded up).

	Entities (amount)	Employee (fte)	Annual turnover (million)	Patients (million)
Academic Medical Centres	7 (25)	88.000 (25)	11.000 (25)	1.26 (25)
Top Clinical Hospitals	27 (26)	81.000 (26)	8.100 (26)	4.59 (27)
General Hospitals	41 (26)	53.000 (26)	6.600 (26)	3.73 (27)

### 3.2 Political and legal context

Between 1983 and 2007, Dutch AMCs were founded, adopting the organisational structure that is currently recognised (integrated university-hospital relationship). These AMCs differ from top clinical and general hospitals in that they have been assigned three public functions in addition to that of a general hospital function: (1) providing tertiary care (2) conducting (bio) medical scientific research and (3) offering medical education and training. In 1992 the legal framework for Dutch AMCs went into effect, as part of a complete renewal of the Higher Education Act (WHW, “Wet op Hoger Wetenschappelijk Onderwijs”) (28). The WHW established the role and functioning of AMCs in healthcare, education, and research, as well as their relationships with universities. This Act requires the establishment of a so-called Staff Committee, an advisory body to the Executive Board consisting of all medical department heads who typically also hold full-time professor positions. Since 2006, the Health Insurance Act (1) and the Health Care Market Regulation Act (29) have been in force, these laws created more market forces in healthcare. Under these laws, AMCs must compete with other healthcare providers to obtain production quotas for curative care. In addition, all healthcare providers, including AMCs, must negotiate with healthcare insurers and demonstrate what they do and at what price and quality. The law distinguishes between care left to market forces and functions that require special funding because of their public nature. These public functions give AMCs an exceptional position compared to general and top clinical hospitals. In a letter to the House of Representatives, the health minister and the state secretary for education stress that innovation and development of top referral care cannot be left to market forces, because then the public interest of sufficient supply and quality is not guaranteed. The Dutch Healthcare Authority and the Dutch Competition Authority must ensure that AMCs do not impede market forces (e.g., by using their additional resources to compete unfairly with other institutions in basic care) (30).

In 1998, the health minister and the state secretary for education reiterated the need to maintain, improve and further develop the top referral function in academic hospitals in addition to the public functions (31). These tasks are all the more challenging as the different functions of AMCs have different sources of funding, making it difficult in practice to distinguish which money flow is used for which task and to what extent the money flows contribute to the public functions of AMCs (32) (Supplementary File 5).

The AMCs (united under the Dutch Federation of University Medical Centres), and the health minister started the ROBIJN project (33). This project aimed to establish definitive criteria that could delineate the characteristics of an academic patient and enable the qualification of top-tier referral care. Using these labels, it is possible to determine which organisation has an academic patient population and is therefore eligible for a financial contribution (34). In other words, this was an instrument that also had to show stakeholders that AMCs are different and that they deserve additional public funding. In 2014 the minister of health and the minister of economic affairs wrote a report outlining the unique position they believe AMCs occupy in the healthcare landscape. However, the ministers felt that the AMCs should make a greater effort to reach mutual agreements on the distribution and concentration of care (35). In 2019, the health minister underscored the importance of AMCs in a letter addressed to the House of Representatives. He emphasised that the social responsibilities of AMCs justify their current financial and strategic advantages over other hospitals. Furthermore, he assigned AMCs the responsibility of effecting change to enhance their distinctiveness and efficiency, thereby ensuring the long-term sustainability of healthcare expenditure (3).

Since their inception, the position and unique role of AMCs have regularly been the subject of political discussions. After 40 years, the AMCs have apparently failed to parry these discussions. However, their position in the Dutch healthcare landscape is viewed with more than mere criticism. This is partly attributed to the complex interrelationships between the government, insurers, AMCs, medical faculties and professional interest groups. Additionally, the lack of substantial changes over decades, as evident in the history of AMCs, contributes to this perspective. In our study, we characterise the latter as muddling through or incrementalism.

### 3.3 Interviews

The interviews show that cooperation among AMCs and between AMCs and other stakeholders is hampered by a number of issues. Supplementary File 6 contains a detailed overview of the main findings by issue.

#### 3.3.1 Conflict of interest

All participants noted that conflicts of interest between AMCs prevented collaboration and decisions that might benefit Dutch society as a whole. AMC directors indicated that if they had to choose, they felt a responsibility to put the interests of their own organisation first. They also noted that collaboration with regional hospitals was hampered by differences in values, vision, and organisational culture. Other interviewees underlined this and noted that these differences are often the unspoken reason (undercurrent) why collaboration between these parties is difficult. The remarks made by Participant 1, the chairman of the board of directors of an AMC, were illustrative: *‘But before we get there (one AMC instead of the current eight, red.), the management style we are used to will not work. That is certainly a cultural thing. And it also has to do with favours and people’*. ‘*I have seen the battle between Utrecht, Rotterdam, Amsterdam, and Leiden over the children’s hospital (concentration of pediatric oncology red.). We are in each other’s way. Between dream and deed stand laws and practical objections’*.

#### 3.3.2 Organisational complexity

Several bottlenecks related to organisational complexity were mentioned. Most interviewees felt that although the AMC’s relationship with a university distinguishes it from regional hospitals such collaboration also increases organisational complexity and hampers efficiency. Four participants felt that the strong influence of academic medical specialists and professional groups hinders the governance of the AMC. Supervision by different government agencies (e.g., the Ministry of Health and the Ministry of Education) was seen as inefficient and a reason behind the complexity of AMC governance.

#### 3.3.3 Governance

Most participants mentioned that greater directive guidance from the government could stimulate collaboration among healthcare organisations. They also view such guidance as not only desirable but obligatory. Participant 6 explained this perspective, stating ‘*Politics is ultimately responsible for the public interest. But we have placed so many responsibilities externally (..) that at the moment politicians are hardly in a position to take back the reins’.*

Participants perceived the traditionally strong Dutch consensus culture as difficult and time-consuming leading to delays, or even failures, in implementing changes. Participant 5, chairman of the AMC board of directors, noted: *‘.. and as administrators among yourselves, you may think that something should be done in a certain way, but the question is whether your employees, and especially the medical specialists, agree with that’*. However, the same culture of consensus can simultaneously facilitate broad support.

#### 3.3.4 Competition

Most respondents believe that competition improves the quality of care and research. However, all participants felt that competition between AMCs and between AMCs and regional hospitals is currently so fierce that it hinders collaboration and decision-making for the benefit of society. Some even spoke of collaborations being disrupted due to the lack of trust caused by competition among AMCs and regional hospitals. Participant 7 said, *‘We are attacked from two sides: we have to give away regular care to regional hospitals, but on the other hand we compete with them for high-complexity care and the academic funds that go with it. If you do not stop this, I think an undesirable situation will arise, where valuable resources are spread too thinly, making it impossible to invest in certain spearheads. (..). And of course AMCs must be monitored for efficiency and there must be some incentive, but it must not endanger the survival of the current healthcare system with the pyramid referral system in which smaller hospitals refer to larger hospitals and these refer to AMCs as a last resort’*.

#### 3.3.5 Collaboration

There is unanimous agreement that collaboration is an important strategy for AMCs to improve the quality of care and research. However, participants indicated that constructive collaboration depends on personal relationships, which they identified as a vulnerable aspect of establishing sustainable collaborative partnerships. Indeed, cooperation between AMCs and regional hospitals is characterised by different interests, focuses, and organisational cultures. Adding to the complexity, the financial system emphasises outcome-based financing and individual performance rather than collective performance. Most participants perceived this as a barrier to successful cooperation. Finally, participants mentioned that competition between hospitals was fierce and had existed for a long time. Consequently, collaboration based on trust and mutual benefits is not self-evident.

#### 3.3.6 Concentration of high-complexity care

Concentration of high-complexity care in AMCs could be beneficial, according to all participants. They stated that the concentration of tertiary care does not necessarily have to take place in all AMCs but could be accommodated in two or three AMCs/centres. A quote from participant 3, former chairman of the board of directors of a health insurance company: *‘Everyone thinks everything is important. Setting priorities is difficult, setting posteriorities is even more difficult’.* The most frequently given arguments for concentration were increased quality of care and increased efficiency as a result of economies of scale. Some interviewees thought that more concentration of complex care should go hand in hand with more decentralisation of regular care. The AMC board members were unanimous in their opinion that a certain level of less complex care was of the utmost importance for education and research as students learn most from common diseases, not rare diseases, and research into more “common” diseases has greater social impact.

#### 3.3.7 Public and regional role

All participants agreed that AMCs have a public and regional role. However, respondents raised doubts regarding the prioritisation of these roles by the AMCs. The three board members of the participating AMCs faced the dilemma that on the one hand they manage large organisations with a large number of employees and on the other hand they are supposed to serve the public interest, which can sometimes be conflicting. Four interviewees stressed the importance of working on health and social issues specific to their respective regions. Considering the regional context, it is evident that issues will vary across each AMC.

#### 3.3.8 Tripartite function

Participants unanimously agreed that, along with their relationship with the university, the tripartite function differentiated AMCs from other hospitals or healthcare organisations. However, the integration of these three core tasks (healthcare, research, and education) within a single organisation makes AMCs inefficient. In this context, one participant wondered whether the different core tasks necessarily have to function within one organisation or whether they could be separate, cooperating entities, which may mitigate some of the inefficiencies.

#### 3.3.9 Market regulation

Since the implementation of the Health Insurance Act in 2006, market forces have been introduced into the healthcare system. The system is based on regulated competition between health insurers and healthcare providers, with the objective of delivering optimal care to citizens at the most favourable cost. However, all interviewees expressed a unanimous belief that genuine market regulation is lacking, at best resulting in a quasi-market or semi-regulated market. Four of them argued that market forces should not apply to healthcare. According to them, market forces do not provide incentives to improve cooperation among healthcare providers.

## 4 Discussion

Dutch AMCs fulfil public tasks within one organisation including (highly complex) patient care, education, training, and research. This leads to a complex governance of seemingly incompatible interests and has called into question the effectiveness and transparency of AMCs’ governance since their inception. Our study identifies nine issues affecting the effectiveness of governance in Dutch AMCs. Constructive cooperation among AMCs and between AMCs and other hospitals is negatively affected by: (1) negative undercurrents and unspoken issues due to conflicts of interest, (2) organisational complexity due to the relationship with a university and with academic medical specialists, (3) lack of sufficient government direction, (4) competition between AMCs due to perverse systemic incentives, (5) different interests, focus and organisational culture, (6) concentration of care, which does not always lead to enhanced quality and efficiency as the provision of less complex care is of utmost importance for education and research, (7) the infeasibility of public and regional functions of an AMC, (8) the inefficiency of three core tasks within the same organisation and, (9) healthcare market regulation.

Our study shows that stakeholders perceive AMCs as inherently technically inefficient. However, this does not necessarily imply inefficiency in terms of allocation and quality of care.

### 4.1 Complex governance

AMCs are considered one of the most complex organisations in the world due to their tripartite mission, the absence of a formal hierarchy, and the presence of public duties (36). AMC leaders struggle with this complexity, as evidenced by the multitude of solutions they deploy. These leaders often unsuccessfully look for solutions to organisational changes and business models (37–39). Prior to this research, several scholars have drawn attention to the importance of considering the number of AMCs to meet contemporary challenges. DeAngelis juxtaposes Darwin’s survival of the fittest with Kropotkin’s emphasis on collaboration, arguing that collaboration prevails when a common goal is present. She sees a solution in reducing the number of AMCs by a national decision. She quotes Fein, who is of the same opinion and also emphasizes the collective responsibility of AMCs in addressing these issues (40, 41). Porter et al. elaborated on this in 2015 (42). In their article, Porter et al. asked whether mergers are necessary to build up the required volume, or whether the organisation should expand via partnerships and affiliations. They call upon leaders to make strategic choices, also regarding density and size. This perspective certainly applies to the Dutch context, where the distinctiveness and competitive positioning of each Dutch AMC compared to the others is limited (39). The number of AMCs in the Netherlands has been a subject of ongoing debate in opinion magazines for years. Various proposals have emerged ranging from decreasing the number of AMCs (whether through mergers or not) to considering the establishment of just one AMC with nationwide coverage.This latter suggestion involves the creation of academic departments in other hospitals, thereby leaving room for research and teaching. The discussions and trends surrounding the allocation of tasks among AMCs and the concentration of highly specialised patient care, such as cardiac surgery and paediatric oncology, are ongoing (43, 44).

### 4.2 Collaboration

Our research shows that successful collaboration among AMCs, and between AMCs and other hospitals, is hampered by mutual competition and undercurrents/unspoken issues due to perverse systemic incentives. Since the start of the previous decade, the main strategy to improve effectiveness seems to have shifted from organisational integration to networking and increased collaboration (45). However, various studies show that the expected benefits of these initiatives are usually not realised (46–48). Actual implementation often fails to materialise after deciding to integrate or collaborate, partially because of the effects of market regulation. However, although it remains undisputed in public discussion, our research reveals a strong undercurrent (unspoken issues) that has a negative influence on successful collaboration, and thus, the effectiveness of AMCs. Participants openly mentioned conflicting interests, perverse financial incentives, institutional pride, mistrust, and competition as hindrances. This undercurrent affects network strategy, as it plays a role in the relationship between AMCs and regional hospitals. In the Netherlands, AMC leaders face hindrances due to market-driven incentives, which impede their ability to establish a shared healthcare vision and adopt a collaborative approach to intricate governance challenges.

### 4.3 Critical junctures in sight?

Based on the research findings, the question is justified as to whether it is still effective, feasible, sustainable, or desirable for the eight Dutch AMCs to continue performing the total portfolio of hospital care, tertiary care (bio), medical research, education, training, and other societal tasks. Indeed, this leads to increasing wicked governance problems due to multiple stakeholders and multiple conflicting demands. This question is supported by Baumgartner and Jones’ punctuated equilibrium theory. In their 2009 publication, they argue that they chose the terminology of punctuated equilibrium because it conjures up the image of stability being interrupted by drastic changes in a system. Systems can be stable without necessarily being in equilibrium, which is why they do not want to claim that all periods of stability are signs of equilibrium; they can simply be the result of the absence of external disturbances (49). Years of muddling through and searching for solutions to wicked governance problems and effectiveness, invites a radical rethinking about the governance of AMCs in the Netherlands. The recent statements from the current health minister shed light on the attitudes of healthcare administrators and the abundance of healthcare organisations in the country. Leaders of healthcare organisations should be more aware and act in the greater interest of healthcare (50). The situation in healthcare calls for a paradigm shift. The pressing demands for care and the shortage of staff are significant issues, underscoring the importance of healthcare parties ceasing their competition and being compelled to collaborate. The Scientific Council for Government Policy (WRR) released a report in 2021, stating that the quality and accessibility of care is coming under increasing pressure due to an ageing population, the emergence of new care technology and the increase in the number of chronically ill people. In order to ensure the long-term financial, human resource, and social sustainability of healthcare, the WRR advocates limiting the growth of care and making better choices regarding care prioritisation (51). The pressure of rising healthcare costs, increasing labour shortages, and the rising number of patients with multiple chronic conditions is being felt in the healthcare sector. This burning platform is further fuelled by massive inflation, the energy crisis, and the aftermath of the two-year pandemic. Under these circumstances, the Integral Care Agreement (“Integraal Zorgakkoord”) was recently concluded in the Netherlands. This agreement calls on all parties to “*bring about a radical change in the Dutch healthcare system and also in society’s perspective on healthcare*” (52). However, our research has shown that a number of conditions must be met before such delicate discussions about (radical) change can take place. Ideally, the government should take the lead and create conditions that foster mutual trust and common interests between AMCs on the one hand and between AMCs and other hospitals on the other. This should lead to an environment, a marketplace, where AMC leaders can discuss change and also feel safe to put the common interest above the interest of their own organisation. Interviewees in our research are open to a more guiding role of the government. Following the punctuated equilibrium theory, the government can act as facilitator of external disturbances to ensure a new equilibrium.

### 4.4 Limitations

The limited number of participants included in this study may have influenced the assessment of the results (53). However, the interviewees were selected because they held positions where they had integral knowledge of the subject matter (21). As this was an exploratory study on a broad topic, unstructured interviews were chosen (54). Unstructured interviews can take unexpected turns, making data collection and analysis challenging. Interviews may each have a different focus on the topic, making comparison difficult and, relevant topics may go undiscussed or the opposite, irrelevant topics may be discussed. This study aimed to mitigate these challenges by applying coding through thematic analysis. Coding was carried out by one author (MT) and a second author (EC) provided feedback. The same method was applied to create subthemes as overarching themes. Thus, coder bias was reduced. A thematic analysis should be viewed with caution. This form of analysis can be subjective as it predominantly relies on the researchers’ judgment. Additionally, there is a risk that certain themes may be overlooked due to the emphasis on identifying larger or overarching themes.

## 5 Conclusion

We hypothesised that radical change could provide a solution to the current incrementalism in AMCS and explored the conditions under which such changes could or could not be achieved. Our hypothesis that radical change offers a solution to the current incrementalism in AMCs could not be adequately explored. Indeed, our exploration of the conditions under which radical change could take place revealed that there are currently factors at play that make implementing radical reforms in healthcare difficult, if not impossible. Organisational complexity, the absence of mutual trust and shared interests, and distorted systemic incentives hinder a substantive debate on the forms of cooperation and the position or number of AMCs in the Netherlands. Incumbent AMC leaders find it difficult to subordinate the interests of their own organisations to the broader interests.

Greenwood and Hinings have developed a model to understand organisational change (11). They identify two internal pressures for change. First, the presence of groups that are dissatisfied with the way their interests are represented within an organisation. These groups link the prevailing organisational structure (which shapes the distribution of advantages and disadvantages) to what they are dissatisfied with at the time when alternatives are available.

Our study found that a certain level of dissatisfaction with the current organisation of AMCs is related to the organisational structure. However, the explicit prompting of the discussion topic of the alternative of one AMC did not give decisive results regarding the relationship dissatisfaction and organisational structure. Greenwood and Hinings indicate that dissatisfaction does not guide change. To this end, they identify a second crucial source of pressure, referred to as the “pattern of value commitments”. They identify four generic patterns of value commitment: (1) status quo (all groups are committed to the existing organisation); (2) indifferent (groups are neither committed nor against); (3) competitive (some groups support the current organisation, while others prefer an articulated alternative); (4) reforming (all groups are against the current organisation and prefer an articulated alternative). Based on our research findings, we position Dutch AMCs in the pattern of competitive commitment. After all, the opinions of the various stakeholders clearly show competitive elements. According to Greenwood and Hinings, radical change is possible if there is competitive value commitment but because competitive change implies the presence of resistance, competitive commitment will be associated with evolutionary change (incrementalism).

If there is any internal pressure to change, radical change can only occur in conjunction with two factors that facilitate radical change. First, Greenwood and Hinings see a reciprocal relationship between power dependencies and value commitments. Radical change in a situation with a competitive pattern of commitment is unlikely unless those in privileged positions and with power are in favour of the proposed change. Power dependencies enable or suppress radical organisational change. Second, the ability to manage the transition process from one organisation to another. This means having sufficient understanding of the new conceptual destination, having the skills and competences needed to function in that new destination, and having the ability to manage how to achieve that destination. High capacity for action is associated with radical change. The interviewees in our study could hardly imagine a possible change in the organisation, let alone a change in the Dutch healthcare landscape where there would be only one AMC. Nor did they come up with alternative suggestions. This indicates that the questions concerning the necessary skills and management for this change were not addressed at all.

In summary, if we contrast the results of our research with Greenwood and Hinings’ precipitating and enabling dynamics, we can conclude that a radical change debate is unlikely in the short term. Although interviewees signal that the current organisational structure is flawed, these signals are not expressed with the same intensity by all stakeholders. There are opposing views on how AMCs should organise and relate to other stakeholders. Some interviewees even talk about conflicting interests, fierce competition and mistrust. This is linked to power dependencies that suppress radical organisational change. None of the interviewees show great capacity for action. All these observations confirm a situation and culture of incrementalism and little to none breeding ground for radical change.

Ideally, the government should take the lead and create conditions that foster mutual trust and common interests between AMCs and between AMCs and other hospitals. This should lead to an environment in which AMC leaders can discuss change and feel safe putting the common interest above the interest of their own organisation. Following punctuated equilibrium theory, the government can act as a facilitator of external disturbances to ensure a new equilibrium.

Knowledge about the current research topic is still in its infancy. It has been found that there is still little scientific literature available on the governance of academic medical centres (55). At the same time, it is known that the governance problems of European AMCs are perceived as similar (56). Therefore, this study may be of interest to countries in similar circumstances that want to start a discussion about a change. A robust follow-up study on this topic is warranted which could involve obtaining responses from a larger and more diverse set of respondents.

## Data availability statement

The original contributions presented in the study are included in the article/Supplementary material, further inquiries can be directed to the corresponding author.

## Ethics statement

All respondents signed an informed consent form before completing the questionnaire. The Research Ethics Committee of the Radboud University confirmed that this study was conducted in compliance with relevant legislation governing research ethics review, such as Medical Research involving Human Subjects Act and Medical Treatment Contracts Act (file number 2022–13898). Consequently, the study received ethical approval from the Research Ethics Committee.

## Author contributions

All authors contributed to conception and design of the study. EC and MT conducted interviews, and coded and analysed data. EC wrote the first draft of the manuscript. All authors contributed to the article and approved the submitted version.
