# Supplementary material for: Academic medical centres in the Netherlands: muddling through or radical change?
Source: Front Public Health. 2024 Jan 4;11:1252977. doi: 10.3389/fpubh.2023.1252977 (PMC10794299; doi:10.3389/fpubh.2023.1252977)
Supplement: Supplementary file 1 [file Table_1.docx]

**SUPPLEMENTARY FILE 1 COREQ (Consolidated criteria for reporting qualitative studies): 32-item checklist**

^Tong A, Sainsbury P, Craig J. Consolidated criteria for reporting qualitative research (COREQ): a 32-item checklist for interviews and focus groups.^ *^International Journal for Quality in Health Care^*^. 2007. Volume 19, Number 6: pp. 349 – 357^

| **No. Item** | **Guide questions/description** | **Reported on Page #** |
| --- | --- | --- |
| **Domain 1: Research team and reﬂexivity** |  |  |
| *Personal Characteristics* |  |  |
| 1. Interviewer/facilitator | Which author/s conducted the interview or focus group? | Page 3 |
|  | *(EC) (MT)* |  |
| 2. Credentials | What were the researcher’s credentials? E.g. PhD, MD |  |
|  | *EC; LLM civil and criminal law*  *MT; MD, Sixth-year medical student*  *PJ; PhD, Parttime Professor of Fiscally Sustainable Healthcare Systems at the Medical Faculty of Radboud University*  *Hubert Berden (HB); MD, PhD, Parttime Professor of Organisation of Hospital Care at the Radboud University/ Radboudumc.* |  |
| 3. Occupation | What was their occupation at the time of the study? |  |
|  | *EC; managing director Operating Rooms, Anesthesiology, Pain & Palliative Medicine*  *MT; Sixth-year medical student Radboud university*  *PJ; Parttime Professor of Fiscally Sustainable Healthcare Systems at the Medical Faculty of Radboud University and parttime Science Officer of the Dutch Ministry of Health, Welfare and Sport*  *HB; Parttime Professor of Organisation of Hospital Care at the Radboud University/ Radboudumc and, Chairman of the Board of the ETZ (Elisabeth-TweeSteden Hospital)* |  |
| 4. Gender | Was the researcher male or female? | N/A |
| 5. Experience and training | What experience or training did the researcher have? |  |
|  | *EC; completed scientific university education, supplemented by various PhD training courses and workshops in preparation for qualitative research*  *MT; within the framework of university medical training, several research traineeships abroad*  *PJ: extensive experience in research and in supervising PhD students*  *HB: extensive experience in research and in supervising PhD students* |  |
| *Relationship with participants* |  |  |
| 6. Relationship established | Was a relationship established prior to study commencement? | N/A |
| 7. Participant knowledge of the interviewer | What did the participants know about the researcher? e.g. personal goals, reasons for doing the research | Supplementary File 3 |
|  | *Prior to the interview, the interviewees received written information about the background of the interviewers and that this research is part of a PhD track.* |  |
| 8. Interviewer characteristics | What characteristics were reported about the inter viewer/facilitator? e.g. Bias, assumptions, reasons and interests in the research topic | Supplementary File 3 |
|  | *All participants received written information about the purpose of the research. Prior to the interview, the interviewees received written information about the background of the interviewers and that this research is part of a PhD track.* |  |
| **Domain 2: study design** |  |  |
| *Theoretical framework* |  |  |
| 9. Methodological orientation and Theory | What methodological orientation was stated to underpin the study? e.g. grounded theory, discourse analysis, ethnography, phenomenology, content analysis | Page 3 |
|  | *Grounded Theory Approach* |  |
| *Participant selection* |  |  |
| 10. Sampling | How were participants selected? e.g. purposive, convenience, consecutive, snowball | Page 3 |
|  | *Purposive sampling; focus on the selection of participants possessing characteristics associated with the research study* |  |
| 11. Method of approach | How were participants approached? e.g. face-to-face, telephone, mail, email | Page 3 |
|  | *Interviews were preferably conducted in-person, but also by using video or telephone due to restrictions in connection with the coronapandemic.* |  |
| 12. Sample size | How many participants were in the study? | Page 3, Supplementary File 2 |
|  | *7 participants were included* |  |
| 13. Non-participation | How many people refused to participate or dropped out? Reasons? | None |
|  | *Out of 7 participants who were asked to participate, 6 initially agreed. One wished not to participate due to the sensitive nature of the subject. However, this participant did agree to participate after a final inquiry of interest and additional information on the subject. As a result, 7 participants were included.* |  |
| *Setting* |  |  |
| 14. Setting of data collection | Where was the data collected? e.g. home, clinic, workplace | Page 3 |
|  | *The data was collected during the interview. There were three variants: home or workplace of the interviewee or digital and then the researchers were based at the Radboudumc.* |  |
| 15. Presence of non-participants | Was anyone else present besides the participants and researchers? | N/A |
| 16. Description of sample | What are the important characteristics of the sample? e.g. demographic data, date | Page 3 |
|  | *The respondents in this study were selected and considered to be representative towards the main strategic issues of AMCs. Respondents from different organisations, with different functions and different perspectives on the healthcare landscape were asked to participate in order to broaden the scope of the subject of this study.* |  |
| *Data collection* |  |  |
| 17. Interview guide | Were questions, prompts, guides provided by the authors? Was it pilot tested? | Page 3 |
|  | *Unstructured interviews were chosen because structured interviews or questionnaires might unintentionally steer the interviewees.*  *The opening question in the interviews was deliberately stimulating in order to challenge the interviewees to a sharp discussion. We asked for their (radical) ideas about the Dutch healthcare landscape, that is if there were only one AMC instead of the current eight.*  *A written invitation and explanation was sent to the interviewees prior to the interview along with the informed consent form.* |  |
| 18. Repeat interviews | Were repeat inter views carried out? If yes, how many? | N/A |
| 19. Audio/visual recording | Did the research use audio or visual recording to collect the data? | Page 3 |
|  | *The interviews were transcribed using an intelligent verbatim transcription method:* ATLAS.ti 8.4.20. |  |
| 20. Field notes | Were ﬁeld notes made during and/or after the interview or focus group? | N/A |
| 21. Duration | What was the duration of the interviews or focus group? | Page 3 |
|  | *The length of the interviews varied between approximately 45 and 60 minutes* |  |
| 22. Data saturation | Was data saturation discussed? | Page 3 |
|  | *Due to the open-ended structure of the interviews, there is a possibility that not all topics were covered. The authors agree that key themes emerged in this study.* |  |
| 23. Transcripts returned | Were transcripts returned to participants for comment and/or correction? | Page 3 |
|  | *All interviewees were invited to receive the transcripts for comment. Only one interviewee felt the need to do so. However, this participant did not wish to make any changes to the transcript.* |  |
| **Domain 3: analysis and ﬁndings** |  |  |
| *Data analysis* |  |  |
| 24. Number of data coders | How many data coders coded the data? | Page 4 |
|  | *The coding was prepared by one author (MT) and provided with feedback by a second author (EC). The same pattern was applied to the creation of the sub-themes as the overarching themes. In this way, the risk of bias on the part of the coder was reduced.* |  |
| 25. Description of the coding tree | Did authors provide a description of the coding tree? | Supplementary File 4, Page 4 |
|  | *A total of 97 codes were derived from 7 transcripts. These codes were grouped into 14 sub-themes. These codes were then organised into 9 overarching themes to establish connections between the different codes* |  |
| 26. Derivation of themes | Were themes identiﬁed in advance or derived from the data? | Supplementary File 4, Page 4 |
|  | *Codes were created using an inductive coding strategy and then were thematically analysed wherein loose codes were grouped into sub-themes and overarching theme. For each sub-theme and overarching theme, quotations were marked in the transcripts to elaborate on the context or meaning.* |  |
| 27. Software | What software, if applicable, was used to manage the data? | Page 4 |
|  | *ATLAS.ti 8.4.20.* |  |
| 28. Participant checking | Did participants provide feedback on the ﬁndings? | Page 4 |
|  | *Participants were invited to provide feedback on the transcription. None of the interviewees made any changes.* |  |
| *Reporting* |  |  |
| 29. Quotations presented | Were participant quotations presented to illustrate the themes/ﬁndings? Was each quotation identiﬁed? e.g. participant number | Throughout the manuscript |
|  | *A total of seven quotations are included throughout the manuscript, italicised with the designation of the participant number.* |  |
| 30. Data and ﬁndings consistent | Was there consistency between the data presented and the ﬁndings? | Supplementary File 6, Pages 4-9 |
|  | *Match Supplementary File 6 and Results section* |  |
| 31. Clarity of major themes | Were major themes clearly presented in the ﬁndings? | Pages 4-9 |
|  | *The most common and recurring themes from those discussions are included in the results* |  |
| 32. Clarity of minor themes | Is there a description of diverse cases or discussion of minor themes? | Pages 4-9, Supplementary File 6 |
|  | *In the Results section, there is also a description of themes that were, for example, mentioned by only one or two participants. Supplementary File 6 gives an overview of the most striking themes.* |  |
