# Supplementary material for: Academic medical centres in the Netherlands: muddling through or radical change?
Source: Front Public Health. 2024 Jan 4;11:1252977. doi: 10.3389/fpubh.2023.1252977 (PMC10794299; doi:10.3389/fpubh.2023.1252977)
Supplement: Supplementary file 2 [file Table_2.docx]

**SUPPLEMENTARY FILE 2 Overview of participants** **their (former) occupation and their respective organisation**

| **PARTICIPANT** | **(FORMER) OCCUPATION** | **ORGANISATION** |
| --- | --- | --- |
| 1 | Chairman of the Board of Directors | Academic Medical Centre |
| 2 | Strategic Advisor | Academic Medical Centre |
| 3 | Former chairman of the Board of Directors | Health Insurance Company |
| 4 | Chairman of the Board of Directors | Health Insurance Company |
| 5 | Chairman of the Board of Directors | Academic Medical Centre |
| 6 | Former Minister | Government |
| 7 | Director | Umbrella organisation AMCs |
