# Supplementary material for: Academic medical centres in the Netherlands: muddling through or radical change?
Source: Front Public Health. 2024 Jan 4;11:1252977. doi: 10.3389/fpubh.2023.1252977 (PMC10794299; doi:10.3389/fpubh.2023.1252977)
Supplement: Supplementary file 3 [file Table_3.docx]

**SUPPLEMENTARY FILE 3 Introduction To The Interviews**

The following written invitation and explanation (translated from Dutch into English) was sent to the interviewees prior to the interview along with the informed consent form.

**Governance of one UMC in a Changing Healthcare Landscape**

- Ester Cardinaal LLM, managing director anaesthesiology, pain and palliative medicine and operating theatres, Radboudumc
- Martijn Tjan, medical student and intern Radboudumc

**Background**

In addition to the objectives regarding the core tasks patient care, research and education, the University Medical Centres (UMCs) are challenged by a changing healthcare landscape.

- More and more emphasis is being placed on the public-societal role that the UMCs should play while the government does not take the lead.
- The UMCs compete with each other on various dossiers
- Their unique position is under discussion
- Financial resources are under pressure
- The population is ageing rapidly, co-morbidity is increasing and expensive technology is making more and more treatments possible
- UMCs must make agreements with each other about the distribution of care and resources.

In 2014 the Minister of Health, Welfare and Sport published a position paper on the role of the UMC. In his letter of July 2019 the then Minister of Health, Welfare and Sport followed this up with several assignments for the UMCs.

Ester Cardinaal has become fascinated by the pressure exerted on the UMCs by various sides and is curious to know how this is responded to by the directors of the UMCs and which strategies are successful. Therefore, in 2018 she started a PhD research project on the subject of the strategies of UMCs and their place in the healthcare landscape. This research consists of several parts including several literature studies, an international comparison by means of a survey and interviews.

**Interview topics**

If there was only one UMC in the Netherlands, what would that mean for, e.g.

- organisation of care
- organisation of a UMC and
- the financing of care?

A tantalising question that is, for the time being, purely hypothetical.

We would like to talk to you in an open interview to find out what you think about it. What opportunities and threats do you see? What does the relationship with the universities look like? Does this have advantages or disadvantages for the three core tasks? What do the hospitals and other care institutions around one UMC look like? Does it lead to superspecialisation or not? Will it create more or fewer opportunities for research? What will happen to training? Will it make healthcare cheaper or more expensive? And so on.

**Interview format**

- An open interview can be compared to a brainstorming session.
- Given its creative nature, the interview should preferably take place in person, subject of course to the coronation rules. But if you prefer, video calling is also a good alternative.
- The interviews will be recorded, after permission, and edited at a later date.
- If you wish, you can give feedback on both the transcript and the analysis.
- No names are used in the analysis, but positions are used.
- Only the intern and the supervisors have access to the recording.
- After transcribing and analysing, the recording will be deleted.
- If quotes are used in the final article, this will be done anonymously.
- Participation is completely voluntary and you can always withdraw from the study without giving any reasons.
