# Supplementary material for: Academic medical centres in the Netherlands: muddling through or radical change?
Source: Front Public Health. 2024 Jan 4;11:1252977. doi: 10.3389/fpubh.2023.1252977 (PMC10794299; doi:10.3389/fpubh.2023.1252977)
Supplement: Supplementary file 4 [file Table_4.docx]

**SUPPLEMENTARY FILE 4 ORGANISATION OF CODES**

In total, 94 codes were derived from 7 transcriptions. These were grouped in 14 subthemes (only 4 different subthemes shown in figure for demonstration purposes). Last, the codes and their accompanying subthemes were grouped in 9 overarching themes (3 shown here).

**All the codes, not yet organised**

**Codes organised in subthemes; e.g. ‘financial’**

**Codes organised in overarching themes to make it easier to find connections between subthemes**

Theme 1

Theme 2

Theme 3
