# Supplementary material for: Academic medical centres in the Netherlands: muddling through or radical change?
Source: Front Public Health. 2024 Jan 4;11:1252977. doi: 10.3389/fpubh.2023.1252977 (PMC10794299; doi:10.3389/fpubh.2023.1252977)
Supplement: Supplementary file 5 [file Table_5.docx]

**SUPPLEMENTARY FILE 5 Funding structure Dutch Academic Medical Centres**

| **CORE FUNCTION** | **MONEY LENDER** | **MONEY FLOW** | **RECIPIENT** |
| --- | --- | --- | --- |
| Patientcare | health insurers | Premium funded care | AMC |
|  | ministry of health | Policy rule Availability Contribution Academic Care (“Beschikbaarheidsbijdrage Academische Zorg”) | AMC |
| Training | ministry of health | Policy rule Availability Contribution Training Fund (“Beschikbaarheidsbijdrage Opleidingsfonds”) | AMC |
| Training / education 1st and 2nd phase / basic doctor | ministry of education | State contribution | Medical Faculty + AMC |
| Research | ministry of health | Policy rule Availability Contribution Academic Care - Development Innovation (“Beschikbaarheidsbijdrage Academische Zorg – Ontwikkeling Innovatie”) | Medical Faculty + AMC |
| Training | ministry of education | Workplace function | Medical Faculty + AMC |
| Research | (Semi) governments, funds, foundations, businesses | 2nd, 3rd, 4th money flow* | Medical Faculty + AMC |
| Valorisation | State contribution | Faculty contribution | Medical Faculty + AMC |

^*The first flow of funds: comes directly from the Ministry of Education, Culture and Science. The second flow of funds: comes from independent public organisations such as the NWO (Netherlands Organisation for Scientific Research). The third flow of funds: is project-related funding, often from private institutions. Fourth flow of funds: funds from private organisations with a profit motive.^

^* Source: Rathenau Institute Chiong Meza CS, J. van; Jonge, Jos de. De Nederlandse Universitair medische centra. Feiten & Cijfers 12 2014.^
