# Supplementary material for: Academic medical centres in the Netherlands: muddling through or radical change?
Source: Front Public Health. 2024 Jan 4;11:1252977. doi: 10.3389/fpubh.2023.1252977 (PMC10794299; doi:10.3389/fpubh.2023.1252977)
Supplement: Supplementary file 6 [file Table_6.docx]

**SUPPLEMENTARY FILE 6 MAIN FINDINGS PER THEME (**INTENDED FOR COLOR REPRODUCTION)

| **THEME** | **STATEMENT** | **AMC** | **AMC** | **UMBRELA ORGANISATION** | **STRATEGIC ADVISER** | **HEALTHCARE INSURER** | **HEALTHCARE INSURER** | **GOVERNMENT** |
| --- | --- | --- | --- | --- | --- | --- | --- | --- |
| **UNDERCURRENT** | AMCs promote their own interests before the public interests |  |  |  |  |  |  |  |
|  | Underlying values, vision and organisational culture hinders collaboration |  |  |  |  |  |  |  |
| **ORGANIZATIONAL COMPLEXITY** | The different tripartite functions differ greatly in governance, culture, values, business models, et cetera |  |  |  |  |  |  |  |
|  | The relationship between AMCs and their university creates organization complexity |  |  |  |  |  |  |  |
|  | The influence of medical specialists and other professionals within an AMC complicate organization |  |  |  |  |  |  |  |
| **GOVERNANCE** | A more directive style of the government is needed to stimulate or even oblige collaboration |  |  |  |  |  |  |  |
|  | The Dutch consensus culture complicates decision making and collaboration |  |  |  |  |  |  |  |
| **COMPETITION** | Currently, competition prevents collaboration and decisions benefiting society |  |  |  |  |  |  |  |
|  | Outcome financing is inducive to competition in contrast to collaboration |  |  |  |  |  |  |  |
|  | AMCs and other hospitals have competed for a long time, inciting distrust and hampering collaboration |  |  |  |  |  |  |  |
| **COLLABORATION** | Collaboration improves quality of care and research |  |  |  |  |  |  |  |
|  | Collaboration is built upon people, making it fragile |  |  |  |  |  |  |  |
| **CONCENTRATION** | High complexity care should be concentrated more than it currently is |  |  |  |  |  |  |  |
|  | Certain technological/expensive infrastructure should be concentrated more than it currently is |  |  |  |  |  |  |  |
|  | Concentration might lead loss of expertise in the other AMCs |  |  |  |  |  |  |  |
|  | AMCs should stop doing most regular care |  |  |  |  |  |  |  |
|  | There should always be at least 2 locations, to have friendly competition and redundancy |  |  |  |  |  |  |  |
| **PUBLIC AND REGIONAL ROLE** | AMCs do not seriously see society’s interest as a priority |  |  |  |  |  |  |  |
|  | AMCs are working at health-related issues in their region |  |  |  |  |  |  |  |
|  | AMCs should have a leading role in their region |  |  |  |  |  |  |  |
| **TRIPARTITE FUNCTION** | More of the research and education could be off-loaded to other hospitals |  |  |  |  |  |  |  |
|  | Having the tripartite function in 1 organisation creates added value |  |  |  |  |  |  |  |
| **MARKET REGULATION** | There are true market forces at play in the Dutch healthcare sector |  |  |  |  |  |  |  |
|  | Market forces mainly benefits healthcare/AMCs |  |  |  |  |  |  |  |
|  | Market forces sometimes prevent collaboration |  |  |  |  |  |  |  |

**_Legend:_**

- _Not discussed/no opinion given._
- _Positive stance towards statement._
- _Negative stance towards statement._
